# New multilocus sequence typing scheme for *Enterococcus faecium* reveals sequential outbreaks of vancomycin-resistant *E. faecium* ST1162 and ST610 in a Japanese tertiary medical center

**DOI:** 10.1128/spectrum.02131-24

**Published:** 2024-12-10

**Authors:** Masaki Karino, Masashi Yanagihara, Tetsuya Harada, Megumi Sugo, Mizuki Karino, Hirofumi Ohtaki, Hiroyuki Hanada, Toru Takano, Masaya Yamato, Shigefumi Okamoto

**Affiliations:** 1 Department of Clinical Laboratory and Biomedical Sciences, Laboratory of Medical Microbiology and Microbiome, Division of Health Sciences, Osaka University Graduate School of Medicine, Suita, Osaka, Japan; 2 Department of Clinical Laboratory, Rinku General Medical Center, Izumisano, Osaka, Japan; 3 Department of Medical Technology, Faculty of Health Sciences, Kansai University of Health Sciences, Sennan-gun, Osaka, Japan; 4 Division of Microbiology, Osaka Institute of Public Health, Osaka, Japan; 5 Departments of General Internal Medicine and Infectious Diseases, Rinku General Medical Center, Izumisano, Osaka, Japan; University of Maryland School of Medicine, Baltimore, Maryland, USA

**Keywords:** *Enterococcus faecium*, vancomycin-resistant, multilocus sequence typing, pulsed-field gel electrophoresis, outbreak

## Abstract

Vancomycin-resistant *Enterococcus faecium* (VREfm) is a major nosocomial pathogen, and molecular epidemiological tools are crucial for controlling its spread. Pulsed-field gel electrophoresis (PFGE) is still used in clinical laboratories despite the increased accessibility of whole-genome sequencing (WGS). As PFGE equipment is no longer commercially available, clinical laboratories need alternative tools. Highly standardized multilocus sequence typing (MLST) is one option. However, the original MLST scheme for *E. faecium*, designed in 2002, showed inconsistencies with WGS-based typing. Therefore, the new Bezdíček MLST scheme, which offers more accurate genetic similarity based on genome-wide data, has recently been proposed. To clarify its clinical utility in analyzing nosocomial VREfm transmission, we compared both MLST schemes with PFGE using 68 VREfm isolates collected during an outbreak at a Japanese tertiary medical center in 2019. PFGE analysis identified nine clusters among the 68 strains, including two predominant clusters. The original scheme identified five sequence types (ST_O_s), of which 82.4% (56/68) were ST_O_192. The Bezdíček scheme identified nine sequence types (ST_B_s), subdividing the original ST_O_192 into ST_B_1162 (30/56), ST_B_610 (25/56), and ST_B_895 (1/56). Simpson’s index of diversity values were 0.635, 0.317, and 0.648 for PFGE, the original scheme, and the Bezdíček scheme, respectively. Combining the Bezdíček scheme with admission records provided clearer outbreak visualization, indicating that two distinct ST_B_s independently caused sequential outbreaks. With high discriminatory power comparable with PFGE and global availability, the Bezdíček scheme is a practical and valuable tool for controlling nosocomial VREfm infections in clinical laboratories.

**IMPORTANCE:**

In areas where vancomycin-resistant *Enterococcus faecium* is common, hospital-acquired infections pose a considerable threat to patients’ lives owing to treatment difficulties. Although whole-genome sequencing-based typing has logically become the new reference standard and its accessibility is growing, many clinical laboratories still lack the fundamental resources to exploit its full potential. Limited availability of the traditional pulsed-field gel electrophoresis test in clinical settings has necessitated the use of alternative tools such as Bezdíček multilocus sequence typing. This study tested the clinical utility of the Bezdíček scheme by comparing it with pulsed-field gel electrophoresis. Designed using Czech isolates, this scheme showed comparable discriminatory powers with the traditional method for geographically distinct Japanese isolates and clearly visualized outbreaks. These findings suggest that the Bezdíček scheme is a potential alternative to pulsed-field gel electrophoresis for identifying hospital transmission of vancomycin-resistant *Enterococcus faecium* in clinical laboratories.

## INTRODUCTION

Vancomycin-resistant *Enterococcus faecium* (VREfm), now frequently multidrug-resistant, is among the most challenging causes of healthcare-associated infections (1). Molecular epidemiological tools are important for controlling nosocomial transmissions and must be readily available in clinical laboratories. Pulsed-field gel electrophoresis (PFGE) for *E. faecium*, introduced in 1991 after the emergence of VREfm (2), remains in use today despite the increased accessibility of genome analysis. However, PFGE analysis has limitations, including procedural complexity and issues with data incompatibility (3). Furthermore, as PFGE equipment is no longer commercially available, there is an urgent need for alternative tools that can be implemented in clinical laboratories.

In 2002, multilocus sequence typing (MLST) for *E. faecium* was developed, enabling the classification of evolutionary lineages by comparing the sequences of seven housekeeping genes in the original scheme (4). However, its discriminatory power is not high, making it unsuitable for epidemiological studies of genetically related isolates, such as those involved in nosocomial outbreaks. However, it is widely used in international comparative studies because of its analytical clarity. In fact, epidemiological studies using the original scheme have helped reveal the global spread of the clonal complex-17 (CC17) strain, which has adapted to the hospital environment (5). Furthermore, various sequence types (STs) within CC17 lacking the *pstS* gene, one of the target genes of MLST, have been identified as endemic worldwide (6–9). These STs can affect the accuracy of MLST analysis. The original scheme was developed using genomic information of *E. faecalis* and MLST data of other bacterial species, as the whole genome information of *E. faecium* was not available at that time. Consequently, phylogenetic evolutionary analysis using the original scheme does not always align with whole-genome analysis (10, 11).

To improve the accuracy of genetic similarity assessment, a new MLST scheme, called the Bezdíček scheme, comprising eight new loci was proposed in 2023 based on genomic analysis of numerous *E. faecium* isolates (12). A total of 194 isolates from a Czech hospital were subjected to whole-genome analysis over a prolonged period (June 2017 to July 2022) and were subdivided into 23 STs using the Bezdíček scheme, expanding from the nine STs identified using the original scheme (12). However, no detailed descriptions of nosocomial transmission between patients or follow-up were provided, leaving the utility of the new scheme during outbreaks unclear. In another report of a VREfm outbreak at a pediatric ward in Brazil, all five analyzed strains were assigned to the same STs using the original and Bezdíček schemes (13). Therefore, the discriminatory power of the new scheme in outbreak analysis remains uncertain.

To address the gap in the literature, this study aimed to validate the clinical utility of the Bezdíček MLST scheme using VREfm isolates from outbreaks at a Japanese tertiary medical center, representing a geographical departure from the previous studies.

## MATERIALS AND METHODS

### Isolate collection and ethics approval

This retrospective study analyzed VREfm isolated from patients at a tertiary medical center in Southern Osaka, Japan, in 2019. An outbreak in early 2019 led to simultaneous VREfm detection in hospitalized patients. During this outbreak, 8,019 VRE screening tests on stools or rectal swabs, as well as follow-up surveys for carriers, were performed. Screening tests and routine culture tests were used to detect vancomycin non-susceptible *E. faecium* 213 and 11 strains, respectively. The study used 68 strains, the first VREfm isolate from each patient, excluding 154 duplicate cases and two unstored cases. Forty-seven strains were isolated from stools, 19 strains from rectal swabs, one from a wound (R-VRE-029), and one from midstream urine (R-VRE-039), with no infection diagnosed. The carrier of R-VRE-039 was repeatedly identified using stool samples following the isolation of VREfm from urine. However, the stool of carrier of R-VRE-029 was not screened, owing to their poor general condition prior to VRE transmission. Regardless of the number of days between admission and detection, all strains were included in this study to facilitate the analysis of their different epidemiological relationships. This study was approved by the Rinku General Medical Center Ethics Committee (approval number: 2023-011). Informed consent was waived, given the use of anonymized retrospective data.

### Microbiological tests

VREfm was isolated on Trypticase Soy Agar with 5% Sheep Blood (BD, Franklin Lakes, NJ, USA) incubated overnight at 35°C. For VRE screening tests, stool or rectal swab samples were plated on CHROMagar VRE (CHROMagar, Paris, France) and incubated at 35°C for 24 h. The MicroScan WalkAway system (Beckman Coulter, Brea, CA, USA) was used for identification and antimicrobial susceptibility at the time of isolation, following the procedure manual. The stored strains were tested for vancomycin and teicoplanin susceptibility using the E-test (bioMérieux, Marcy l'Etoile, France) and Mueller Hinton II Agar (BD) in accordance with Clinical and Laboratory Standards Institute standard methods (https://clsi.org/).

### DNA extraction

The stored strains were cultured overnight in Brain Heart Infusion Broth (Eiken Chemical, Tokyo, Japan) and then moderately diluted in saline solution. Total DNA was extracted using magLEAD 6gC and MagDEA Dx SV (Precision System Science, Chiba, Japan), following the manufacturer’s instructions.

### Detection of *van* and *ddl* genes

The presence of glycopeptide resistance genes (*vanA* and *vanB*) and the species-specific *ddl* gene in VREfm isolates was detected using multiplex polymerase chain reaction (PCR), following a modified procedure from a previous study (14). The primers used are listed in Table S1. Briefly, a 20 µL reaction mixture was prepared using the KAPATaq Extra PCR Kit (Kapa Biosystems, Wilmington, MA, USA) according to the manufacturer’s instructions. The PCR conditions were as follows: 94°C for 2 min, followed by 25 cycles at 94°C for 15 s, 54°C for 15 s, 72°C for 45 s, and 72°C for 1 min. Amplified fragments were confirmed using 1.5% agarose gel electrophoresis.

### PFGE

PFGE analysis was also performed as previously described (15). Briefly, total DNA embedded in agarose plugs was digested with SmaI, and the resulting fragments were separated via PFGE using a CHEF-DRIII apparatus (Bio-Rad Laboratories, Hercules, CA, USA). The band patterns were analyzed using BioNumerics software (Applied Maths, Sint-Martens-Latem, Belgium). Dendrograms for cluster analysis were generated with the following conditions: Dice coefficient, represented by the unweighted pair group method with arithmetic mean (UPGMA), with 1.0% optimization and 1.0% tolerance. Clusters were classified based on 85% similarity.

### MLST

The target genes for MLST were amplified using primer sets from previous studies (4, 12). The primers used are listed in Table S1. A 20 µL reaction mixture was prepared using the KAPATaq Extra PCR Kit. For the original MLST, the PCR conditions were as follows: 94°C for 4 min, followed by 35 cycles of 94°C for 30 s, 52°C for 1 min, 72°C for 1 min, and 72°C for 7 min. For the Bezdíček scheme, the annealing temperature was changed to 60°C, with other conditions remaining the same. After confirmation by agarose gel electrophoresis, the PCR products were purified enzymatically with Exonuclease I (New England Biolab [NEB], Ipswich, MA, USA) and Shrimp Alkaline Phosphatase (NEB) following the manufacturer’s instructions. The purified DNA fragments were sequenced by Fasmac (Kanagawa, Japan) and Eurofins Genomics (Tokyo, Japan). The sequences were checked for allele numbers and STs using pubMLST (16). A new allelic sequence (*ddl*-194) was deposited in the DNA Data Bank of Japan (accession no. LC814434), and all MLST data were deposited in the pubMLST database (16). In this study, to avoid confusion regarding the ST numbering, we used ST_O_ and ST_B_ to distinguish between the original and Bezdíček schemes in *E. faecium* MLST. Phylogenetic trees were constructed using the UPGMA method in MEGA11 (17) from the concatenated MLST allele sequences of the STs identified in this study. Genetic linkages between the identified MLST allele profiles were analyzed using the goeBURST Full MST algorithm in PHYLOViZ 2.0 (18).

### Discriminatory power and clustering concordance

Simpson’s index of diversity (19) and the adjusted Wallace index (20) were calculated to determine discriminatory power and clustering concordance, respectively, using the online tool for comparing partitions (http://www. comparingpartitions.info/).

### Investigation of VREfm carriers

The ward transfer history of VREfm carriers was investigated, and the number of days spent in each ward before testing positive was tabulated. Data regarding antacid proton pump inhibitors (PPIs), H2 blockers, potassium-competitive acid blockers (P-CABs), and vancomycin administration prior to VREfm isolation were also collected from medical records.

## RESULTS

### Clinical isolate

**Fig 1:**
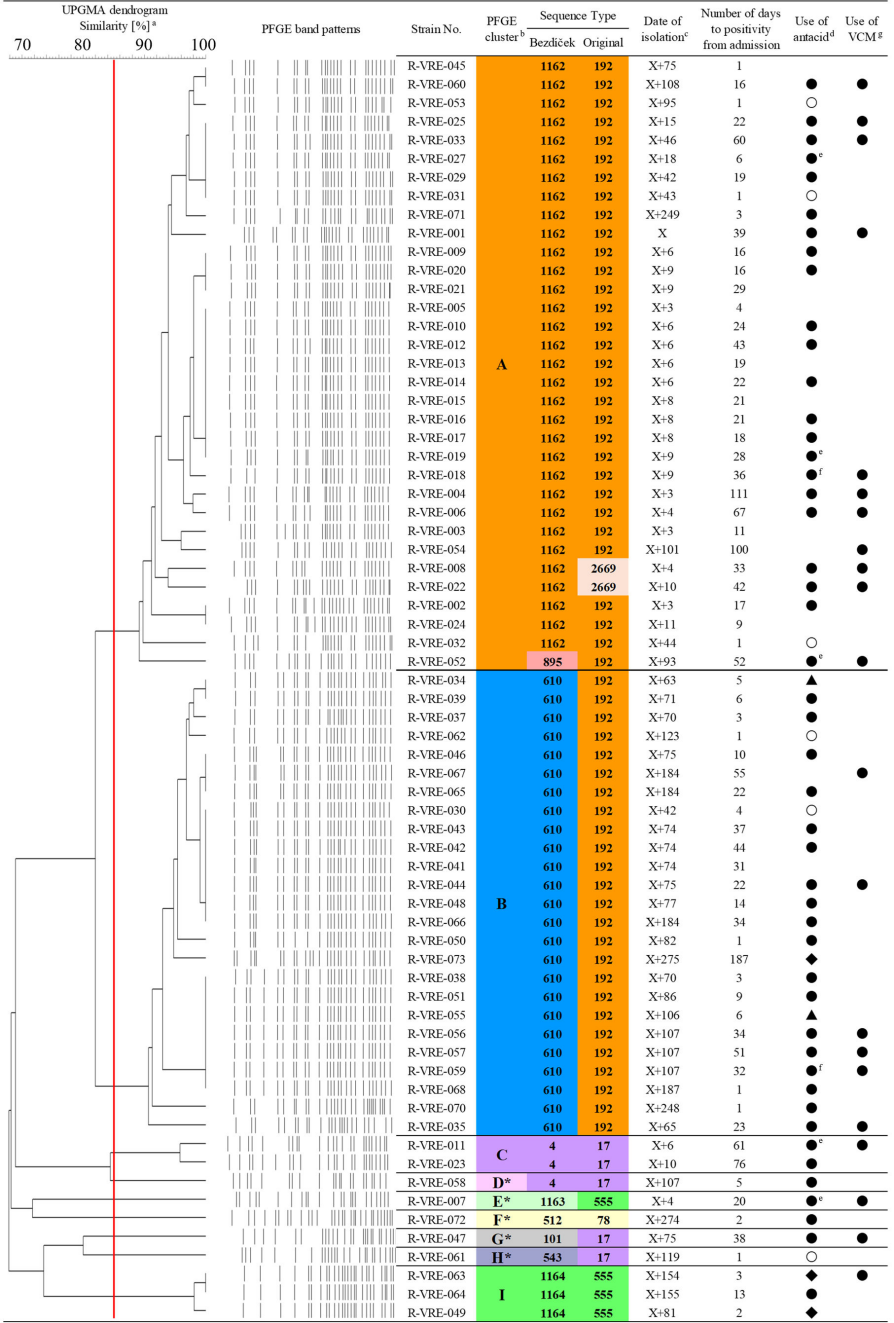
Comparison of ST distribution between the original and Bezdíček MLST schemes based on PFGE clustering analysis of the studied strains. (a) The dendrogram of the unweighted pair group method with arithmetic mean (UPGMA) was derived from the similarity of PFGE band patterns. The red line indicates 85% similarity. (b) Cluster analysis is based on 85% similarity. *: singleton. (c) The date of the first VREfm isolate in 2019 is presented as "day X". (d) The circles, diamonds, and triangles represent the use of proton pump inhibitors (PPIs), H2 blockers, and potassium-competitive acid blockers (P-CABs), respectively. The duration of use prior to the detection of VRE is indicated by colors: black for ≥3 days and white for <3 days. (e) Indicates that PPIs and H2 blockers were used. (f) Indicates that PPIs and P-CABs were used. (g) Indicates that vancomycin was used before the isolation of VRE.

The *vanA* genes were identified in all 68 VREfm isolates, whereas the *vanB* gene was not detected. *E. faecium*-specific *ddl* genes were also detected in all strains, which is consistent with the MicroScan WalkAway System results. The carriers included 45 male individuals aged 41–90 years and 23 female individuals aged 48–95 years. The mean and median ages were 73.8 and 74.0 years, respectively, for both sexes. Eleven cases were detected within 48 h of admission (Fig. 1). Seven individuals (R-VRE-031, -032, -045, -049, -050, -053, and -068) had a history of admission since December 2018, two (R-VRE-061 and -062) were transferred from geriatric facilities, and one (R-VRE-072) was transferred from a neighboring medical institution. The remaining individual (R-VRE-070) had no history of admissions since 2015. Although the minimum inhibitory concentrations (MICs) for vancomycin revealed high resistance in all but one strain, those for teicoplanin exhibited variable results (Fig. S1).

### PFGE analysis

Sixty-eight VREfm strains were subjected to PFGE analysis for reference classification and divided into nine clusters (Fig. 1). The two main clusters, A and B, contained 85.3% (58/68) of the total strains. The remainder consisted of small clusters or singletons. Figure 1 also shows the detection dates and the number of days from admission to VREfm detection, with concentrated isolation periods observed in clusters A and B.

### Original MLST analysis

**Fig 2:**
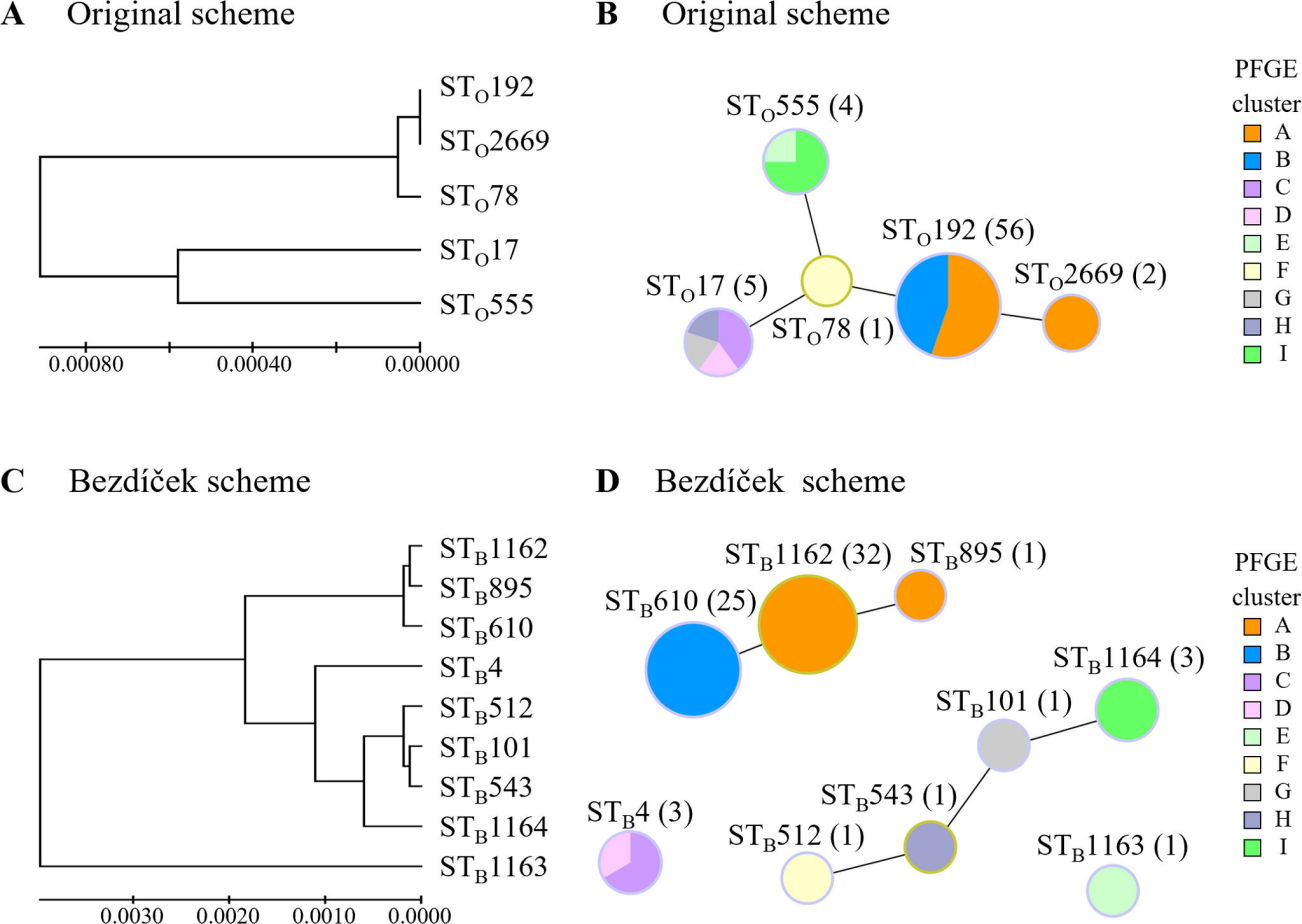
Phylogenetic tree and goeBURST full MST analysis of STs detected using the original and Bezdíček MLST schemes. (**A, C**) UPGMA tree for the concatenated allelic sequences from (**A**) the original scheme and (**C**) the Bezdíček scheme. The evolutionary distances were calculated using the maximum composite likelihood method, measured as the number of base substitutions per site. (**B, D**) Genetic relationships between allele profiles from (**B**) the original and (**D**) Bezdíček schemes, overlaid on PFGE cluster distribution and analyzed using the goeBURST Full MST algorithm at the SLV level. Colors outside ST nodes are shown as group founders in light green and common nodes in light blue. Colors inside ST nodes represent PFGE clusters A to I, as indicated in the figure legend. The size of each node represents the number of isolates on a log-scale, whereas the numbers in parentheses denote the number of isolates in each ST node. The black link represents “without recourse to tiebreak rules.” ST_O_ and ST_B_ indicate the sequence type according to the original MLST and Bezdíček MLST schemes, respectively.

The original MLST scheme identified five ST_O_s: ST_O_192 in 56 strains (82.4%), ST_O_2669 in two (2.9%), ST_O_17 in five (7.4%), ST_O_555 in four (5.9%), and ST_O_78 in one strain (1.5%) (Fig. 1; Table 1). ST_O_2669 closely resembles ST_O_192 but is differentiated by a 21-base duplication in a segment of *ddl*-1, indicating that it is a putative subtype of ST_O_192. Phylogenetic tree analysis of the UPGMA showed that the predominant ST_O_192 was closely related to ST_O_2669 and ST_O_78. However, all ST_O_s were in a single group when classified at an evolutionary distance of 0.001 (Fig. 2A). The goeBURST Full MST analysis at the single-locus variant (SLV) level grouped the five ST_O_s into a single group (Fig. 2B). The group of ST_O_s shared identical alleles at four of the seven loci (Table 1).

**TABLE 1:** List of original MLST scheme allele profiles detected in this study^*a*^

ST_O_	*atpA*	*ddl*	*gdh*	*purK*	*gyd*	*pstS*	*adk*	Number of isolates
192	15	1	1	1	1	7	1	56
2669	15	194	1	1	1	7	1	2
17	1	1	1	1	1	1	1	5
555	4	1	1	1	1	1	1	4
78	15	1	1	1	1	1	1	1

^*a*^ ST_O_, sequence type according to the original multilocus sequence typing scheme.

### Bezdíček MLST analysis

The Bezdíček MLST scheme identified nine ST_B_s: ST_B_1162 in 32 strains (47.1%); ST_B_610 in 25 (36.8%); ST_B_4 in three (4.4%); ST_B_1164 in three (4.4%); and ST_B_895, ST_B_101, ST_B_543, ST_B_512, and ST_B_1163 in one strain each (1.5% each) (Fig. 1; Table 2). Phylogenetic tree analysis of UPGMA showed that the predominant STs, ST_B_1162, and ST_B_610, along with ST_B_895, were closely related to each other but phylogenetically distinct from the other ST_B_s (Fig. 2C). The nine ST_B_s were divided into two groups and two singletons, based on the goeBURST Full MST analysis at the SLV level (Fig. 2D). These groups of ST_B_s shared identical alleles at five or six of the eight loci (Table 2). Similarly, in the UPGMA tree, classification at an evolutionary distance of 0.001 divided the ST_B_s into four groups, consistent with the number of groups formed during goeBURST Full MST analysis.

**TABLE 2:** List of Bezdíček MLST scheme allele profiles detected in this study^*a*^

ST_B_	*copA*	*dnaE*	HP	*mdlA*	*narB*	*pbp2b*	*rpoD*	*urvA*	Number of isolates
1162	2	1	1	3	2	1	5	1	32
610	2	3	1	3	2	1	5	1	25
895	2	1	1	3	3	1	5	1	1
1164	4	1	1	9	2	1	2	2	3
101	4	1	1	1	2	1	2	2	1
543	4	1	1	1	2	1	2	1	1
512	4	1	1	1	2	1	1	1	1
4	1	1	1	1	3	1	1	1	3
1163	1	1	1	1	2	1	11	1	1

^*a*^ ST_B_, sequence type according to the Bezdíček multilocus sequence typing scheme.

### Comparison of molecular epidemiological analysis

The discriminatory power of each method was evaluated using Simpson’s index of diversity, which was 0.635 (95% CI, 0.560–0.710), 0.317 (95% CI, 0.175–0.458), and 0.648 (95% CI, 0.574–0.722) for PFGE, the original scheme, and the Bezdíček scheme, respectively. The Bezdíček scheme exhibited the same discriminatory power as PFGE, which was higher than that of the original scheme. According to the adjusted Wallace index of clustering concordance between methods, the new Bezdíček scheme and PFGE were highly concordant (Table 3). A comparison of the distribution of STs in both MLST schemes with the PFGE classification in Fig. 1 revealed that the distribution of ST_B_s in the Bezdíček scheme closely matched the PFGE clustering. This was also confirmed by the results presented in Fig. 2D, which depicts the Bezdíček scheme allele profiles overlaid on the PFGE cluster distribution and analyzed using the goeBURST full MST algorithm. These observations suggest that the outbreak was caused by two major ST_B_s. In contrast, the original scheme failed to distinguish the two major clusters, identifying them as a single large cluster instead (Fig. 1 and 2; Table S2). Additionally, ST_O_17 and ST_O_555 in the original scheme were distantly distributed among multiple PFGE clusters, a discrepancy resolved by the Bezdíček scheme. These results indicate that the Bezdíček scheme shares the same discriminatory powers and a high clustering concordance with PFGE for epidemiologically relevant strains.

**TABLE 3:** Adjusted Wallace index comparing Bezdíček and original MLST schemes with PFGE^*a*^

	Bezdíček MLST	Original MLST	PFGE
Bezdíček MLST	–	0.764 (0.470–1.000)	0.996 (0.992–1.000)
Original MLST	0.192 (0.119–0.266)	–	0.204 (0.144–0.263)
PFGE	0.941 (0.831–1.000)	0.765 (0.471–1.000)	–

^*a*^ Numbers in parentheses denote the 95% confidence intervals. MLST, multilocus sequence typing; PFGE, pulsed-field gel electrophoresis.

### Visualization of outbreak

**Fig 3:**
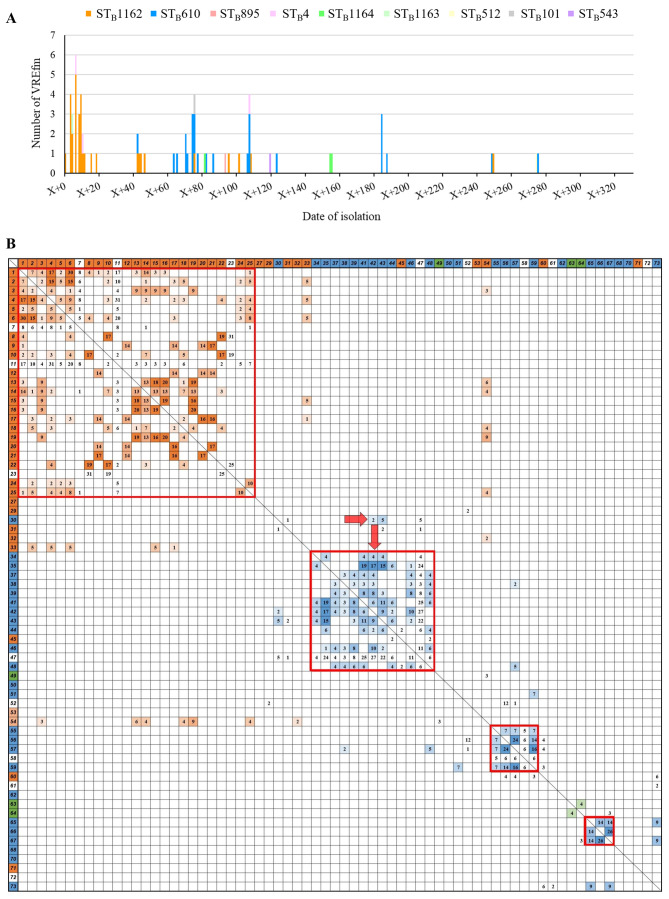
Visualization of the outbreak using the Bezdíček MLST scheme and tracing carriers. (**A**) Distribution of detection dates by ST_B_s from the Bezdíček scheme. ST_B_ indicates the sequence type according to the Bezdíček MLST scheme. (**B**) The number in the outline represents the strain number. The number in each well represents the number of days spent by each carrier in the same ward before positive confirmation. The orange, blue, and green color coding represents contact risks between ST_B_1162, ST_B_610, and ST_B_1164, respectively. The duration in days is represented by four color shades: 1–3 days, 4–7 days, 8–14 days, and 15 or more days. The red-boxed areas indicate the period during which isolation was presumably concentrated owing to horizontal spread within the ward. The red arrows indicate movements that may have triggered transmission in the ward after contact with the carriers.

Figure 3A illustrates the distribution of the date of isolation along with the ST_B_s identified by the Bezdíček scheme. Different ST_B_s were detected consecutively, indicating that they had formed distinct clusters. To visualize horizontal transmission within the ward, the number of days patients were hospitalized in the same ward as VREfm pre-carriers was tabulated (Fig. 3B). The same ST_B_s were observed in clusters among patients in the same ward to identify horizontal transmission (red box in Fig. 3B). Visual observation confirmed the ward cluster shrinking and the outbreak ending owing to infection control measures. Clear visualization also revealed the dynamics of sequential outbreaks caused by different ST_B_s. R-VRE-030 was identified on the fourth day of admission; however, VRE with an identical PFGE band pattern was isolated from two patients who stayed in the same ward during that period, leading to the expansion of the ST_B_610 cluster (Fig. 1 and red arrow in Fig. 3B). In contrast, transmission could not be tracked in patients who were identified with VREfm on admission using a screening test (Fig. 3B). Of patients with a history of previous hospital admission, further investigation revealed that three had a history of contact with carriers during the previous admissions (Table S3). Among them, there was a notable case of suspected transmission from a patient with confirmed negative results on three consecutive VRE culture tests. As shown in Table 4, patient B (R-VRE-033 assigned to ST_B_1162) is presumed to have transmitted VRE to patient A (R-VRE-053 assigned to ST_B_1162) during their initial admission when they shared a room for 5 days.

**TABLE 4:** Timeline of patient events in a case of apparent transmission after negative confirmation^*a*^

Day	Patient A	Patient B
X + 46		R-VRE-033 assigned to ST_B_1162 was isolated
X + 60 to X + 74		Three consecutive negative results after VRE culture tests
X + 63	Admission, negative result after VRE culture test	
X + 77 to X + 81	Shared the same room^*b*^	
X + 81	Discharge	
X + 93		Positive results after VRE culture test
X + 94	Readmission	
X + 95	R-VRE-053 assigned to ST_B_1162 was isolated	

^*a*^ ST_B_, sequence type according to the Bezdíček multilocus sequence typing scheme.

^*b*^ Patient B is presumed to have transmitted VRE to patient A during the 5-day period when they shared a room.

### Administration of antacids and vancomycin

Approximately 76.5% (52/68) of the patients had used antacids for more than 3 days before VREfm detection (Fig. 1). The use of vancomycin was confirmed in 30.9% (21/68) of the patients (Fig. 1).

## DISCUSSION

This study validated the clinical utility of the newly proposed Bezdíček MLST scheme for *E. faecium* during a VREfm outbreak in a medical center. The distribution of ST_B_s classified by the Bezdíček scheme was sufficiently consistent with the phylogenetic tree analysis by PFGE. Conversely, the original scheme showed limited discriminatory power, as the same ST_O_s spanned multiple PFGE clusters. Although it suggested a major outbreak caused by ST_O_192, the Bezdíček scheme and carrier tracing revealed a series of horizontal transmissions by ST_B_1162 and ST_B_610. Moreover, although the discriminatory power of the Bezdíček scheme was moderate (0.648) compared with that from a previous study (0.983) (12), this can be attributed to the analysis of epidemiologically close strain collections. Nonetheless, the results exhibited comparable discriminatory power to PFGE. Generally, MLST is simple, reproducible, and comparable, making it suitable for clinical laboratories. This makes the new Bezdíček scheme a suitable alternative to PFGE as an epidemiological tool for managing nosocomial VREfm infections.

In the primary spread, 25 strains (mainly ST_B_1162) were detected within 16 days of the start of the epidemic. In the second spread, 13 strains (mainly ST_B_610) were detected over a 15-day period starting on day X + 63. Although not evident in the first spread, R-VRE-030 appears to have been the founding strain of the second cluster. It was the ﬁrst ST_B_610 strain isolated on day 4 of admission, and its PFGE band pattern was identical to that of VREfm isolated in November 2018 (Fig. S2). However, the carriers of the two strains were not hospitalized simultaneously, and no clear contact history could be confirmed. Additionally, *Enterococci* can survive on dry surfaces for months or even longer (21), suggesting that these cases might have involved environmental transmission at different times.

Research indicates that the use of PPIs is a factor in the increased risk of nosocomial transmission of VRE and drug-resistant bacteria (22, 23). In this study, 76.5% of patients had a history of taking antacids, such as PPIs, H2 blockers, and P-CABs. As a host-side factor, antacids can increase the pH of the stomach, reducing its barrier function, and allowing bacteria to reach the intestine and persist. Additionally, PPI use can influence the composition of the intestinal microbiota, resulting in a notable increase in the prevalence of *Enterococci* (24). This, in turn, may facilitate the establishment of VRE that has passed through the stomach. However, the present study did not assess non-VRE carriers, limiting risk factor evaluation; therefore, further detailed studies are needed.

In hospitals where VRE is detected, patient isolation and contact precautions are essential to prevent its spread. However, ongoing carrier management is often discontinued after three consecutive negative cultures (25). In the investigated hospital, this criterion for confirming negative results led to subsequent VRE carriage (Table 4). Although VRE can be re-detected after confirmed clearance, especially following antimicrobial therapy (26–28), it is crucial to understand that culture-negative clearance does not indicate complete eradication, but rather that detection levels are below sensitivity thresholds (28). Establishing criteria, such as the addition of a surveillance culture if antimicrobials are administered after a negative confirmation, are recommended. Furthermore, as shown in Table 4, patient B (R-VRE-033 carrier) was on continuous PPI therapy at the time of re-detection. This suggests that repeated entry through the gastric barrier might lead to recurrence, warranting future investigations.

ST_O_192, typed by the original scheme, was the predominant strain and the first isolation reported in Japan according to the pubMLST database (https://pubmlst.org/). Although ST_O_192 is an SLV of ST_O_78, the Bezdíček scheme results suggest lower genetic relatedness than expected (Fig. 2). ST_O_192 was often detected in a European VREfm outbreak around 2010, with many strains carrying the *vanB* gene (29, 30). In contrast, recent ST_O_192 strains in Asia predominantly carry the *vanA* gene (31). Notably, an ST_O_192 isolate from China in 2019 was re-classified as ST_B_895 under the Bezdíček scheme (accession no. JABEXB000000000). Although only one ST_B_895 strain was detected in the present study, it is an SLV of ST_B_1162 and a double-locus variant of ST_B_610, suggesting a potential relationship. The origin of this outbreak requires further investigation; however, a Bezdíček scheme with improved discrimination may clarify this issue.

Whole-genome sequencing (WGS)-based typing has logically become the new reference standard, and its availability is growing, whereas many clinical laboratories, including ours, still lack the fundamental resources to exploit its full potential. Our study showed that the Bezdíček scheme enables outbreak analysis with accuracy comparable with PFGE, remarkably contributing to nosocomial infection control. Additionally, although isolates from the Czech Republic were used to design the new scheme (12), high discriminatory power was also confirmed for geographically unrelated Japanese isolates. In these clinical laboratories that are unable to implement WGS, the Bezdíček MLST scheme is an ideal option for monitoring nosocomial VREfm infections. There are two advantages of performing the Bezdíček MLST scheme analysis. First, this scheme has a more precise resolution of genetic similarity and aligns closely with genome-wide data (12), making it useful for regional and international comparative studies. Second, when implementing WGS in the future, this scheme is compatible with *in silico* MLST analysis, allowing the use of accumulated data without loss. In contrast, *in silico* MLST has also been performed to interpret the results, even in studies using WGS (13, 32, 33). In such cases, it is recommended to use the Bezdíček MLST scheme, which shows more precise classification of genetic similarity than the original scheme.

This study had several limitations. First, the discriminatory power of both MLST schemes was evaluated using PFGE cluster analysis, which does not allow whole-genome analysis. Second, the analyzed strains were isolated from a single medical center in 2019 and cannot be determined for more diverse populations or long-term evaluation.

In conclusion, the Bezdíček MLST scheme shows great potential as a global epidemiological tool owing to its high discriminatory power and accessibility. Its cost-effectiveness, simplicity, and reproducibility make it suitable for routine clinical use. Additionally, this scheme can effectively visualize VREfm outbreaks and assist in infection control without requiring complicated analyses. Our findings show that the Bezdíček MLST scheme can be a suitable alternative to PFGE for real-time analysis of VREfm nosocomial outbreaks in clinical laboratories.

## SUPPLEMENTAL MATERIAL

Supplemental figures and tables spectrum.02131-24-s0001.docx Fig. S1 and S2; Tables S1 to S3.

ASM does not own the copyrights to Supplemental Material that may be linked to, or accessed through, an article. The authors have granted ASM a non-exclusive, world-wide license to publish the Supplemental Material files. Please contact the corresponding author directly for reuse.

## References

[1] Weiner LM, Webb AK, Limbago B, Dudeck MA, Patel J, Kallen AJ, Edwards JR, Sievert DM. 2016. Antimicrobial-resistant pathogens associated with healthcare-associated infections: summary of data reported to the national healthcare safety network at the centers for disease control and prevention, 2011–2014. Infect Control Hosp Epidemiol 37:1288–1301. doi:10.1017/ice.2016.174 27573805 PMC6857725

[2] Miranda AG, Singh KV, Murray BE. 1991. DNA fingerprinting of Enterococcus faecium by pulsed-field gel electrophoresis may be a useful epidemiologic tool. J Clin Microbiol 29:2752–2757. doi:10.1128/jcm.29.12.2752-2757.1991 1757545 PMC270427

[3] van Belkum A, van Leeuwen W, Kaufmann ME, Cookson B, Forey F, Etienne J, Goering R, Tenover F, Steward C, O’Brien F, Grubb W, Tassios P, Legakis N, Morvan A, El Solh N, de Ryck R, Struelens M, Salmenlinna S, Vuopio-Varkila J, Kooistra M, Talens A, Witte W, Verbrugh H. 1998. Assessment of resolution and intercenter reproducibility of results of genotyping Staphylococcus aureus by pulsed-field gel electrophoresis of SmaI macrorestriction fragments: a multicenter study. J Clin Microbiol 36:1653–1659. doi:10.1128/JCM.36.6.1653-1659.1998 9620395 PMC104895

[4] Homan WL, Tribe D, Poznanski S, Li M, Hogg G, Spalburg E, Van Embden JDA, Willems RJL. 2002. Multilocus sequence typing scheme for Enterococcus faecium. J Clin Microbiol 40:1963–1971. doi:10.1128/JCM.40.6.1963-1971.2002 12037049 PMC130786

[5] Willems RJL, Top J, van Santen M, Robinson DA, Coque TM, Baquero F, Grundmann H, Bonten MJM. 2005. Global spread of Enterococcus faecium from distinct nosocomial genetic complex. Emerg Infect Dis 11:821–828. doi:10.3201/1106.041204 15963275 PMC3367597

[6] Carter GP, Buultjens AH, Ballard SA, Baines SL, Tomita T, Strachan J, Johnson PDR, Ferguson JK, Seemann T, Stinear TP, Howden BP. 2016. Emergence of endemic MLST non-typeable vancomycin-resistant Enterococcus faecium. J Antimicrob Chemother 71:3367–3371. doi:10.1093/jac/dkw314 27530751

[7] Hammerum AM, Justesen US, Pinholt M, Roer L, Kaya H, Worning P, Nygaard S, Kemp M, Clausen ME, Nielsen KL, Samulioniené J, Kjærsgaard M, Østergaard C, Coia J, Søndergaard TS, Gaini S, Schønning K, Westh H, Hasman H, Holzknecht BJ. 2019. Surveillance of vancomycin-resistant enterococci reveals shift in dominating clones and national spread of a vancomycin-variable Enterococcus faecium ST1421-CT1134 clone, Denmark, 2015 to March 2019. Euro Surveill 24:1900503. doi:10.2807/1560-7917.ES.2019.24.34.1900503 31456560 PMC6712932

[8] McCracken M, Mitchell R, Smith S, Hota S, Conly J, Du T, Embil J, Johnston L, Ormiston D, Parsonage J, Simor A, Wong A, Golding G. 2020. Emergence of pstS-Null vancomycin-resistant Enterococcus faecium clone ST1478, Canada, 2013–2018. Emerg Infect Dis 26:2247–2250. doi:10.3201/eid2609.201576 32818423 PMC7454069

[9] Saito N, Kitazawa J, Horiuchi H, Yamamoto T, Kimura M, Inoue F, Matsui M, Minakawa S, Itoga M, Tsuchiya J, Suzuki S, Hisatsune J, Gu Y, Sugai M, Kayaba H. 2022. Interhospital transmission of vancomycin-resistant Enterococcus faecium in Aomori, Japan. Antimicrob Resist Infect Control 11:99. doi:10.1186/s13756-022-01136-5 35871001 PMC9308179

[10] Raven KE, Reuter S, Reynolds R, Brodrick HJ, Russell JE, Török ME, Parkhill J, Peacock SJ. 2016. Enterococcus faecium 'a decade of genomic history for healthcare-associated Enterococcus faecium in the United Kingdom and Ireland. Genome Res 26:1388–1396. doi:10.1101/gr.204024.116 27527616 PMC5052055

[11] van Hal SJ, Ip CLC, Ansari MA, Wilson DJ, Espedido BA, Jensen SO, Bowden R. 2016. Evolutionary dynamics of Enterococcus faecium reveals complex genomic relationships between isolates with independent emergence of vancomycin resistance. Microb Genom 2:e000048. doi:10.1099/mgen.0.000048 27713836 PMC5049587

[12] Bezdicek M, Hanslikova J, Nykrynova M, Dufkova K, Kocmanova I, Kubackova P, Mayer J, Lengerova M. 2023. New multilocus sequence typing scheme for Enterococcus faecium based on whole genome sequencing data. Microbiol Spectr 11:e0510722. doi:10.1128/spectrum.05107-22 37306567 PMC10434285

[13] Vasconcelos TM, Mesa D, Rodrigues LS, Medeiros Dos Santos É, Krul D, Siqueira AC, de Abreu RBV, Motta F de A, Conte D, Dalla-Costa LM. 2024. Outbreak of vancomycin-resistant Enterococcus faecium ST1133 in paediatric patients with acute lymphoblastic leukaemia from southern Brazil. J Glob Antimicrob Resist 36:41–44. doi:10.1016/j.jgar.2023.11.005 38000534

[14] Dutka-Malen S, Evers S, Courvalin P. 1995. Detection of glycopeptide resistance genotypes and identification to the species level of clinically relevant enterococci by PCR. J Clin Microbiol 33:24–27. doi:10.1128/jcm.33.1.24-27.1995 7699051 PMC227872

[15] Fujiya Y, Harada T, Sugawara Y, Akeda Y, Yasuda M, Masumi A, Hayashi J, Tanimura N, Tsujimoto Y, Shibata W, Yamaguchi T, Kawahara R, Nishi I, Hamada S, Tomono K, Kakeya H. 2021. Transmission dynamics of a linear vanA-plasmid during a nosocomial multiclonal outbreak of vancomycin-resistant enterococci in a non-endemic area, Japan. Sci Rep 11:14780. doi:10.1038/s41598-021-94213-5 34285270 PMC8292306

[16] Jolley KA, Bray JE, Maiden MCJ. 2018. Open-access bacterial population genomics: BIGSdb software, the PubMLST.org website and their applications. Wellcome Open Res 3:124. doi:10.12688/wellcomeopenres.14826.1 30345391 PMC6192448

[17] Tamura K, Stecher G, Kumar S. 2021. Mega11: molecular evolutionary genetics analysis version 11. Mol Biol Evol 38:3022–3027. doi:10.1093/molbev/msab120 33892491 PMC8233496

[18] Nascimento M, Sousa A, Ramirez M, Francisco AP, Carriço JA, Vaz C. 2017. PHYLOViZ 2.0: providing scalable data integration and visualization for multiple phylogenetic inference methods. Bioinformatics 33:128–129. doi:10.1093/bioinformatics/btw582 27605102

[19] Hunter PR, Gaston MA. 1988. Numerical index of the discriminatory ability of typing systems: an application of Simpson’s index of diversity. J Clin Microbiol 26:2465–2466. doi:10.1128/jcm.26.11.2465-2466.1988 3069867 PMC266921

[20] Severiano A, Pinto FR, Ramirez M, Carriço JA. 2011. Adjusted wallace coefficient as a measure of congruence between typing methods. J Clin Microbiol 49:3997–4000. doi:10.1128/JCM.00624-11 21918028 PMC3209087

[21] Neely AN, Maley MP. 2000. Enterococci staphylococci survival of enterococci and staphylococci on hospital fabrics and plastic. J Clin Microbiol 38:724–726. doi:10.1128/JCM.38.2.724-726.2000 10655374 PMC86187

[22] Chanderraj R, Millar JA, Patel TS, Read AF, Washer L, Kaye KS, Woods RJ. 2019. Vancomycin-resistant Enterococcus acquisition in a tertiary care hospital: testing the roles of antibiotic use, proton pump inhibitor use, and colonization pressure. Open Forum Infect Dis 6:fz139. doi:10.1093/ofid/ofz139 PMC6475592 31024976

[23] Willems RPJ, van Dijk K, Ket JCF, Vandenbroucke-Grauls CMJE. 2020. Evaluation of the association between gastric acid suppression and risk of intestinal colonization with multidrug-resistant microorganisms: a systematic review and meta-analysis. JAMA Intern Med 180:561–571. doi:10.1001/jamainternmed.2020.0009 32091544 PMC7042870

[24] Imhann F, Bonder MJ, Vich Vila A, Fu J, Mujagic Z, Vork L, Tigchelaar EF, Jankipersadsing SA, Cenit MC, Harmsen HJM, Dijkstra G, Franke L, Xavier RJ, Jonkers D, Wijmenga C, Weersma RK, Zhernakova A. 2016. Proton pump inhibitors affect the gut microbiome. Gut 65:740–748. doi:10.1136/gutjnl-2015-310376 26657899 PMC4853569

[25] Banach DB, Bearman G, Barnden M, Hanrahan JA, Leekha S, Morgan DJ, Murthy R, Munoz-Price LS, Sullivan KV, Popovich KJ, Wiemken TL. 2018. Duration of contact precautions for acute-care settings modified title: duration of contact precautions for acute-care settings. Infect Control Hosp Epidemiol 39:127–144. doi:10.1017/ice.2017.245 29321078

[26] Baden LR, Thiemke W, Skolnik A, Chambers R, Strymish J, Gold HS, Moellering RC, Eliopoulos GM. 2001. Prolonged colonization with vancomycin-resistant Enterococcus faecium in long-term care patients and the significance of “clearance” Clin Infect Dis 33:1654–1660. doi:10.1086/323762 11595985

[27] Donskey CJ, Hoyen CK, Das SM, Helfand MS, Hecker MT. 2002. Recurrence of Enterococcus stool colonization during antibiotic therapy. Infect Control Hosp Epidemiol 23:436–440. doi:10.1086/502081 12186208

[28] Henard S, Lozniewski A, Aissa N, Jouzeau N, Rabaud C. 2011. Evaluation of the duration of Enterococcus faecium carriage and clearance during a large-scale outbreak in a region of eastern France. Am J Infect Control 39:169–171. doi:10.1016/j.ajic.2010.07.003 20971530

[29] Bender JK, Kalmbach A, Fleige C, Klare I, Fuchs S, Werner G. 2016. Population structure and acquisition of the Enterococcus faecium ST192 resistance determinant in German clinical isolates of Enterococcus faecium ST192. Sci Rep 6:21847. doi:10.1038/srep21847 26902259 PMC4763178

[30] Lytsy B, Engstrand L, Gustafsson Å, Kaden R. 2017. Time to review the gold standard for genotyping vancomycin-resistant enterococci in epidemiology: comparing whole-genome sequencing with PFGE and MLST in three suspected outbreaks in Sweden during 2013-2015. Infect Genet Evol 54:74–80. doi:10.1016/j.meegid.2017.06.010 28627467

[31] Zhou W, Zhou H, Sun Y, Gao S, Zhang Y, Cao X, Zhang Z, Shen H, Zhang C. 2020. Characterization of clinical enterococci isolates, focusing on the vancomycin-resistant enterococci in a tertiary hospital in China: based on the data from 2013 to 2018. BMC Infect Dis 20:356. doi:10.1186/s12879-020-05078-4 32517758 PMC7285731

[32] Valenza G, Eisenberger D, Voigtländer S, Alsalameh R, Gerlach R, Koch S, Kunz B, Held J, Bogdan C. 2023. Emergence of novel ST1299 vanA lineages as possible cause for the striking rise of vancomycin resistance among invasive strains of Enterococcus faecium at a German university hospital. Microbiol Spectr 11:e0296223. doi:10.1128/spectrum.02962-23 37905844 PMC10848474

[33] Rath A, Kieninger B, Caplunik-Pratsch A, Fritsch J, Mirzaliyeva N, Holzmann T, Bender JK, Werner G, Schneider-Brachert W. 2024. Concerning emergence of a new vancomycin-resistant Enterococcus faecium strain ST1299/CT1903/vanA at a tertiary university centre in South Germany. J Hosp Infect 143:25–32. doi:10.1016/j.jhin.2023.10.008 37852539

