# Supplementary material for: New multilocus sequence typing scheme for Enterococcus faecium reveals sequential outbreaks of vancomycin-resistant E. faecium ST1162 and ST610 in a Japanese tertiary medical center
Source: Microbiol Spectr. 2024 Dec 10;13(1):e02131-24. doi: 10.1128/spectrum.02131-24 (PMC11705941; doi:10.1128/spectrum.02131-24)
Supplement: Supplemental figures and tables — Fig. S1 and S2; Tables S1 to S3. [file spectrum.02131-24-s0001.docx]

**SUPPLEMENTAL MATERIAL**

**Supplemental Figures**

**
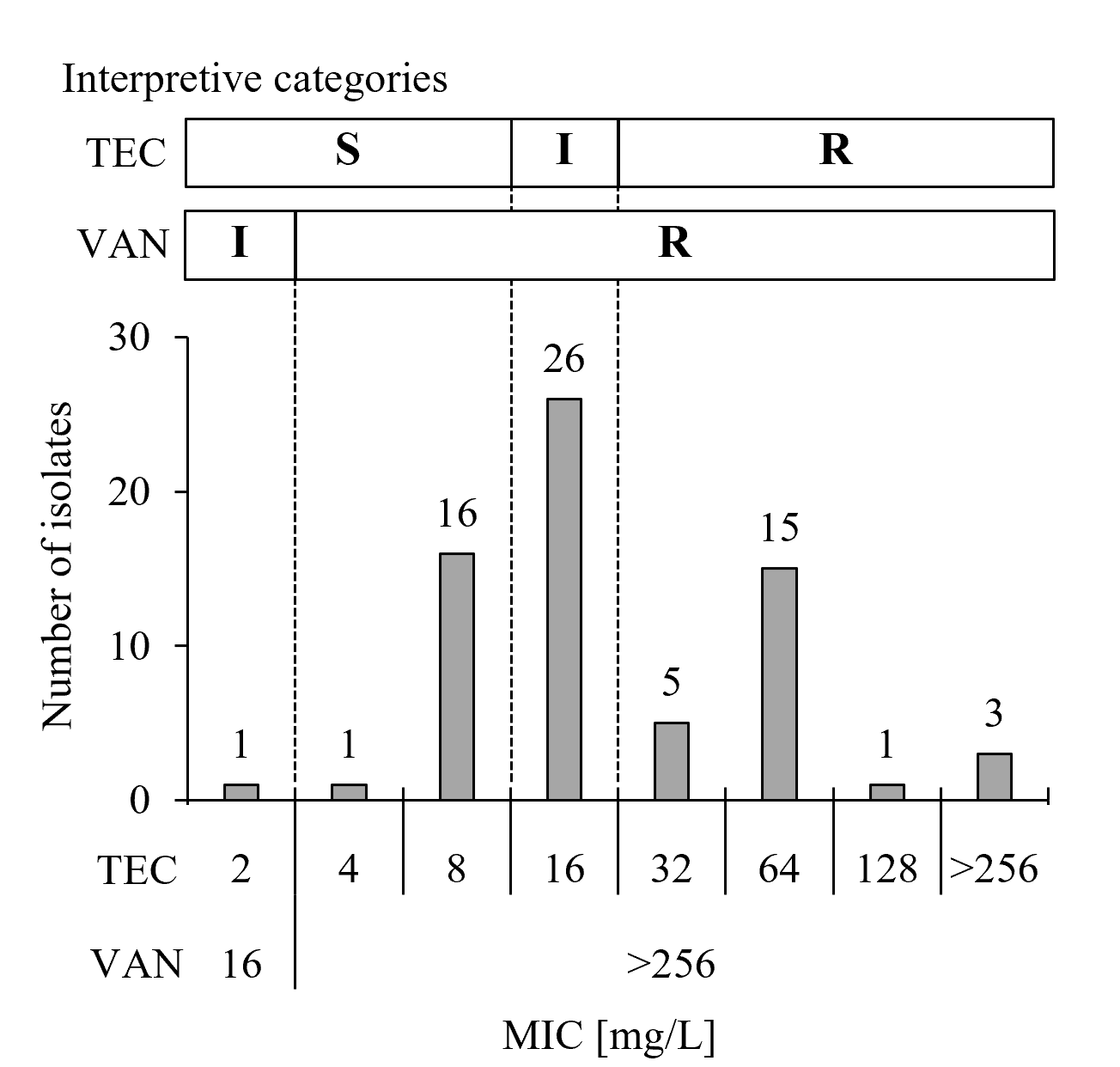
**

**Fig S1** Distribution of minimum inhibitory concentrations for vancomycin and teicoplanin among the isolates.

Interpretive categories are shown according to the Clinical and Laboratory Standards Institute (CLSI) M100 ED34. TEC; teicoplanin, VAN; vancomycin, MIC; minimum inhibitory concentration, S; sensitivity, I; intermediate, R; resistance.

**
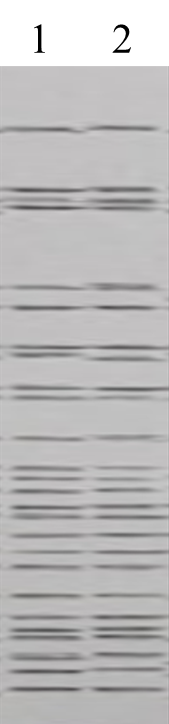
**

**Fig S2** Comparison of PFGE band patterns between R-VRE-030 and the isolate in November 2018.

This image represents the PFGE band patterns obtained by *Sma I* digestion. Lane 1, R-VRE-030; lane 2, the isolate in November 2018. The condition of the PFGE analysis was performed as described in Materials and Methods. PFGE; pulsed-field gel electrophoresis.

**Supplemental Tables**

**TABLE S1** List of primers used for this study.

| Gene | Primer ID | Sequence 5’ to 3’ | Amplicon size (bp) | Reference |
| --- | --- | --- | --- | --- |
| *vanA* | A_1_ | GGGAAAACGACAATTGC | 732 | (14) |
|  | A_2_ | GTACAATGCGGCCGTTA |  |  |
| *vanB* | B_1_ | ATGGGAAGCCGATAGTC | 635 | (14) |
|  | B_2_ | GATTTCGTTCCTCGACC |  |  |
| *ddl _E. faecium_* | F_1_ | GCAAGGCTTCTTAGAGA | 550 | (14) |
|  | F_2_ | CATCGTGTAAGCTAACTTC |  |  |
| *ddl _E. faecalis_* | E_1_ | ATCAAGTACAGTTAGTCTT | 941 | (14) |
|  | E_2_ | ACGATTCAAAGCTAACTG |  |  |
| *atpA* | atpA1 | CGGTTCATACGGAATGGCACA | 556 | (4) |
|  | atpA2 | AAGTTCACGATAAGCCACGG |  |  |
| *ddl* | ddl1 | GAGACATTGAATATGCCTTATG | 465 | (4) |
|  | ddl2 | AAAAAGAAATCGCACCG |  |  |
| *gdh* | gdh1 | GGCGCACTAAAAGATATGGT | 530 | (4) |
|  | gdh2 | CCAAGATTGGGCAACTTCGTCCCA |  |  |
| *purK* | purK1 | GCAGATTGGCACATTGAAAGT | 492 | (4) |
|  | purK2 | TACATAAATCCCGCCTGTTTY |  |  |
| *gyd* | gyd1 | CAAACTGCTTAGCTCCAATGGC | 395 | (4) |
|  | gyd2 | CATTTCGTTGTCATACCAAGC |  |  |

**TABLE S1** (Continued)

| Gene | Primer ID | Sequence 5’ to 3’ | Amplicon size (bp) | Reference |
| --- | --- | --- | --- | --- |
| *pstS* | pstS1 | TTGAGCCAAGTCGAAGCTGGAG | 583 | (4) |
|  | pstS2 | CGTGATCACGTTCTACTTCC |  |  |
| *adk* | adk | TATGAACCTCATTTTAATGGG | 437 | (4) |
|  | adk2 | GTTGACTGCCAAACGATTTT |  |  |
| *copA* | copA FW | TTCTGGAGCTGCTTGATTGC | 518 | (12) |
|  | copA RV | AGTCATTGCGTGTCCATGTG |  |  |
| *dnaE* | dnaE FW | CGCAAATAGCAAGTTTGTGAA | 568 | (12) |
|  | dnaE RV | CGATAAGCGTTGGCTGAAAT |  |  |
| HP | HP FW | CCGTTTGCTGCCTTGATATCA | 582 | (12) |
|  | HP RV | TTCTGAGCCAGTTGATCCAAA |  |  |
| *mdlA* | mdlA FW | AGCAAAGGCAGAGGAATCAG | 464 | (12) |
|  | mdlA RV | CGATTTTTAGAAAATTAGGCTGGT |  |  |
| *narB* | narB FW | GCCGGTGTTCAAATCTCGAT | 403 | (12) |
|  | narB RV | GAGTCGGACGTCAAGCAAAC |  |  |
| *pbp2b* | Pbp2b FW | CATCCGTTTAAAGATGGTAGCAA | 466 | (12) |
|  | Pbp2b RV | CCCATGTTCCTTTTCGCTTA |  |  |
| *rpoD* | rpoD FW | ACGTTCCCGAGTAACACCAA | 527 | (12) |
|  | rpoD RV | GGCATGCAGTTCCTTGACTT |  |  |
| *urvA* | urvA FW | TACGGCGTTCTTTTGGTACA | 694 | (12) |
|  | urvA RV | AAATGATTTTGGTGGCGTTC |  |  |

**TABLE S2** Relationship between sequence types identified using original and Bezdíček MLST schemes in this study.

| ST_O_ by original MLST scheme |  | ST_B_ by Bezdíček MLST schemes | | | | | | | | |
| --- | --- | --- | --- | --- | --- | --- | --- | --- | --- | --- |
|  |  | 1162 | 610 | 4 | 1164 | 1163 | 101 | 895 | 543 | 512 |
| 192 |  | 30 | 25 |  |  |  |  | 1 |  |  |
| 2669*^a^* |  | 2 |  |  |  |  |  |  |  |  |
| 17 |  |  |  | 3 |  |  | 1 |  | 1 |  |
| 555 |  |  |  |  | 3 | 1 |  |  |  |  |
| 78 |  |  |  |  |  |  |  |  |  | 1 |

ST_O_, sequence type according to the original multilocus sequence typing scheme.

ST_B_, sequence type according to the Bezdíček multilocus sequence typing scheme.

*^a^* Subtype of ST_O_192.

**TABLE S3** Contact history during previous admissions of patients with isolated VREfm on readmission.

| Strain No. | ST | Description |
| --- | --- | --- |
| R-VRE-031 | ST_B_1162 | Hospitalized in the same ward with ST_B_1162 carriers (representative strains were R-VRE-001 and -010) at last admission. |
| R-VRE-032 | ST_B_1162 | Before and during the outbreak, there were three hospitalizations and discharges. Hospitalized in the same ward with ST_B_1162 carriers (representative strains were R-VRE-003, -015, and -054). |
| R-VRE-053 | ST_B_1162 | At last admission, shared room with R-VRE-033 (ST_B_1162) carrier with confirmed negative results after three consecutive VRE culture tests. |

ST_B_, sequence type according to the Bezdíček scheme.
